# Healthy Choices Intervention to Address Stigma, Alcohol Use, and Self-Management in Spanish-Speaking Young People With HIV: Protocol for a Pilot Randomized Controlled Implementation Study

**DOI:** 10.2196/103611

**Published:** 2026-07-29

**Authors:** Henna Budhwani, John Waters, Bo Wang, Iván Balán, Sylvie Naar

**Affiliations:** 1 College of Nursing Florida States University Tallahassee, FL United States; 2 Caribbean Vulnerable Communities Coalition Santo Domingo Dominican Republic; 3 UMass Chan Medical School Worcester, MA United States

**Keywords:** HIV, stigma, young people with HIV, emerging adults, alcohol use, motivational interviewing, implementation science, exploration, preparation, implementation, sustainment, EPIS, assessment, decision, adaptation, production, topical experts, integration, training, and testing, ADAPT-ITT, Dominican Republic, community-based organizations, pilot randomized controlled trial

## Abstract

**Background:**

Young people with HIV experience disproportionately poor outcomes across the HIV care continuum, high levels of stigma, and elevated alcohol and other substance use. Healthy Choices is a 4-session intervention that integrates motivational interviewing with brief cognitive-behavioral strategies and has demonstrated a positive impact on HIV stigma, alcohol use, depression, and viral load in prior trials among young people with HIV. Interventions such as Healthy Choices have the potential to improve the HIV care continuum in Caribbean settings. However, no interventions have been adapted in Spanish and pilot-tested to concurrently address stigma, mental health, alcohol use, and viral burden among young people with HIV in the Dominican Republic.

**Objective:**

This study aims to adapt Healthy Choices for Spanish-speaking young people with HIV in the Dominican Republic and pilot-test the adapted intervention with implementation strategies to assess feasibility, acceptability, and a directional signal of its potential impact.

**Methods:**

Guided by the exploration, preparation, implementation, and sustainment framework and the health stigma and discrimination framework, this 36-month study has 3 specific aims. In aim 1, we will conduct focus groups with community representatives and in-depth interviews with young people with HIV to identify barriers, adaptation needs, and implementation strategies. In aim 2, we will use assessment, decision, adaptation, production, topical experts, integration, training, and testing and co-design workshops with community partners and a youth advisory board to contextually translate and adapt Healthy Choices for the Dominican Republic in Spanish, and we will train peer navigators. In aim 3, we will conduct a pilot randomized controlled trial with 45 young people with HIV using a 2:1 allocation to the adapted Healthy Choices intervention (n=30, 67%) or a time-attention control condition (n=15, 33%). Primary outcomes are feasibility and acceptability. Secondary and exploratory outcomes include antiretroviral adherence, viral load, alcohol and other substance use, social support, positive affect, coping, depression, and HIV-related stigma, which will be assessed from baseline to follow-up.

**Results:**

The study period is from March 1, 2026, to February 28, 2029. At the time of manuscript preparation, formative partnership development and key-informant consultation had been completed. The study is positioned to proceed through qualitative exploration, intervention adaptation, peer navigator training, and pilot testing across the 36-month project period. Results from the pilot trial are expected after completion of follow-up assessments.

**Conclusions:**

This protocol describes an implementation science–guided approach to adapting and testing a promising intervention for young people with HIV in a high-priority global setting with translational potential for Spanish-speaking populations in the United States. If the intervention is feasible and acceptable, the study will yield preliminary data for a future full-scale hybrid type 1 effectiveness-implementation trial.

**Trial Registration:**

ClinicalTrials.gov NCT07638618; https://tinyurl.com/5fsdn4fj

**International Registered Report Identifier (IRRID):**

PRR1-10.2196/103611

## Introduction

### Background

Young people with HIV continue to have some of the poorest outcomes across the HIV treatment cascade, including lower rates of viral suppression and greater vulnerability to alcohol and other substance use than older adults [1]. Stigma, alcohol misuse, and poor HIV self-management are synergistic and reinforce one another, contributing to poorer adherence [2,3], reduced engagement in care, and worse mental health [4]. Emerging adulthood is a developmental period marked by autonomy-seeking, identity exploration, and heightened risk-taking [5-7], making developmentally tailored interventions especially important for this age group.

Healthy Choices is a 4-session behavior change communication intervention adapted from the motivational enhancement therapy that integrates motivational interviewing with brief cognitive-behavioral skills [8]. Prior studies have demonstrated that, when delivered with fidelity, Healthy Choices can reduce problematic alcohol use, HIV-related stigma, depression, and viral load among young people with HIV [9]. Healthy Choices can also be delivered by trained community health workers or peer navigators in clinic or community settings [10], making it a strong candidate for adaptation to underresourced and stigma-sensitive contexts.

The Dominican Republic is an important setting for this work [11]. The HIV epidemic in the region has direct translational relevance to the United States, considering the large populations of Dominicans and other Spanish-speaking Caribbean populations in the United States. Stigma remains a major barrier to care for young people with HIV in both countries [12,13]. Prior government-sponsored work in the Dominican Republic found substantial stigma, psychological distress, and substance-based coping among people with HIV. In this context, an intervention that simultaneously addresses alcohol use, HIV self-management, and stigma may improve well-being among young people with HIV while generating knowledge that can directly inform efforts to increase rates of viral suppression in multiple global settings.

### Rationale and Conceptual Framework

This protocol is guided by 2 complementary frameworks. First, the exploration, preparation, implementation, and sustainment (EPIS) framework guides the identification of barriers, contextual determinants, and implementation strategies relevant to adapting Healthy Choices for community settings in the Dominican Republic [14]. The study emphasizes the Exploration and Preparation phases and explicitly engages community-based organizations (CBOs), youth partners, and peer navigators to ensure contextual fit and future scalability.

Second, the health stigma and discrimination framework informs the stigma-reduction logic of the study [15]. In this protocol, stigma is conceptualized as multifaceted and socially structured while also being experienced and managed at the individual level. Healthy Choices is expected to influence fear, motivation, empowerment, and autonomy-supportive decision-making related to medication adherence and alcohol use, thereby reducing internalized and perceived stigma and ultimately improving engagement in HIV care.

### Objective and Specific Aims

The overall objective of this study is to adapt Healthy Choices for Spanish-speaking young people with HIV in the Dominican Republic, cocreate implementation strategies with community partners, and pilot-test the adapted intervention for feasibility, acceptability, and a directional signal of its potential impact.

Aim 1 is to elucidate barriers to delivering Healthy Choices and identify implementation strategies through focus groups with community representatives and in-depth interviews with young people with HIV. Aim 2 is to adapt and contextually translate Healthy Choices using ADAPT-ITT (assessment, decision, adaptation, production, topical experts, integration, training, and testing) [16], co-design workshops, and peer navigator training with competency and fidelity monitoring. Aim 3 is to pilot-test the adapted intervention and implementation strategies in a randomized controlled trial comparing Healthy Choices with a time-attention control condition.

## Methods

### Study Design

This is a multimethod, implementation science–guided pilot study conducted over 36 months. The protocol combines qualitative inquiry, participatory intervention adaptation, peer navigator training, and a pilot randomized controlled trial. Aim 1 uses focus groups and in-depth interviews to identify barriers, priorities, and implementation strategies. Aim 2 uses ADAPT-ITT and co-design workshops to produce a Spanish-language adaptation of the Healthy Choices intervention and accompanying implementation procedures. Aim 3 will pilot-test the adapted intervention in a 2:1 randomized design.

### Setting and Partnerships

The study will be conducted in the Dominican Republic in collaboration with CBO partners. CBO representatives and a youth advisory board (YAB) will contribute to intervention adaptation, recruitment, implementation planning, and interpretation throughout the study.

### Participants and Eligibility

For aim 1 focus groups, community representatives and peer navigators will be eligible if they are aged ≥18 years, identify as peer navigators or community representatives, have at least 4 hours of weekly contact with young people with HIV, and can provide informed consent. For aim 1 interviews, young people with HIV will be eligible if they are aged 18 to 29 years, have HIV, live in the Dominican Republic, and can provide informed consent. For aim 3, young people with HIV will be eligible if they are aged 18 to 29 years, have HIV, speak Spanish, live in the Dominican Republic, self-report experiencing HIV-related stigma in the past 6 months, engage in alcohol misuse, and can provide informed consent.

### Youth Advisory Board

A YAB of 4 to 6 intervention-eligible young people with HIV will be convened to ensure authenticity and contextual relevance. The YAB will meet 2 to 4 times annually, contribute to adaptation decisions, review draft intervention materials, and advise on strategies to maximize engagement, acceptability, and future scale-up.

### Aim 1: Qualitative Exploration of Barriers and Implementation Strategies

Aim 1 will include 2 to 3 focus groups with 4 to 6 community representatives per group and 6 to 8 in-depth interviews with young people with HIV. Interview guides based on the EPIS implementation science framework will focus on barriers to engagement, context-specific stigma experiences, delivery considerations, and implementation strategies to support fidelity, retention, and integration into community-based services. Sessions will be conducted in person in private spaces and audio recorded. Audio files will be stored on encrypted, password-protected servers, transcribed in Spanish, and translated into English. Rapid qualitative analysis matrices will be used to identify adaptation needs [17], cultural nuances, and candidate implementation strategies that will directly inform aim 2.

### Aim 2: Adaptation, Translation, and Interventionist Training

Aim 2 will use ADAPT-ITT to adapt Healthy Choices from English to a contextually appropriate Spanish-language version for young people with HIV in the Dominican Republic. The adaptation process will include an iterative review of aim 1 findings, 2 to 4 in-person co-design workshops with CBO partners and young people with HIV, context-specific translation with back-translation, and review by the YAB and community partners. The intervention is expected to retain its core logic while modifying content, tone, and delivery features to match local priorities and stigma experiences. Candidate implementation strategies may include texting between sessions, phone calls, emailed resources, WhatsApp (Meta Platforms Inc)-based support, and stigma-reduction handouts, depending on stakeholder input and acceptability.

Once the adapted intervention is finalized, accompanying competency and fidelity tools will be revised to match the new intervention content. A matched time-attention control condition focused on diet and nutrition will also be produced using prerecorded materials. For training, 2 to 4 peer navigators selected by community partners will be trained to deliver the intervention during a 2-day workshop conducted in Spanish, followed by 2 to 4 virtual coaching sessions. Competence will be assessed through repeated standardized patient role plays and knowledge assessments. Peer navigators must achieve at least 80% competency to deliver Healthy Choices. Ongoing spot checks using adapted role plays will be used to monitor fidelity.

### Aim 3: Pilot Randomized Controlled Trial

Aim 3 will pilot-test the adapted intervention and implementation strategies in 45 young people with HIV randomized in a 2:1 ratio to Healthy Choices (n=30, 67%) or a time-attention control condition focused on diet and nutrition (n=15, 33%). Randomization will occur after informed consent is obtained by a trained research assistant, using a prespecified, Excel (Microsoft Corp)-based allocation program. Specifically, a random number generator will allocate 30 (67%) participants to the intervention group (coded as 1) and 15 (33%) participants to the control group (coded as 0). After informed consent is collected, participants will be entered into the allocation file and preassigned according to their randomized allocation. The randomization schedule will be maintained by the principal investigator and will not be accessible to interventionists (trained peer navigators). Allocation will be disclosed after participants have been fully onboarded; therefore, interventionists and participants will be unblinded at that point. Thus, outcome assessors will not be blinded. To receive the intervention or control condition, participants will be linked either to a trained peer navigator for Healthy Choices or to a study consultant who will deliver the prerecorded control condition.

**Figure 1:**
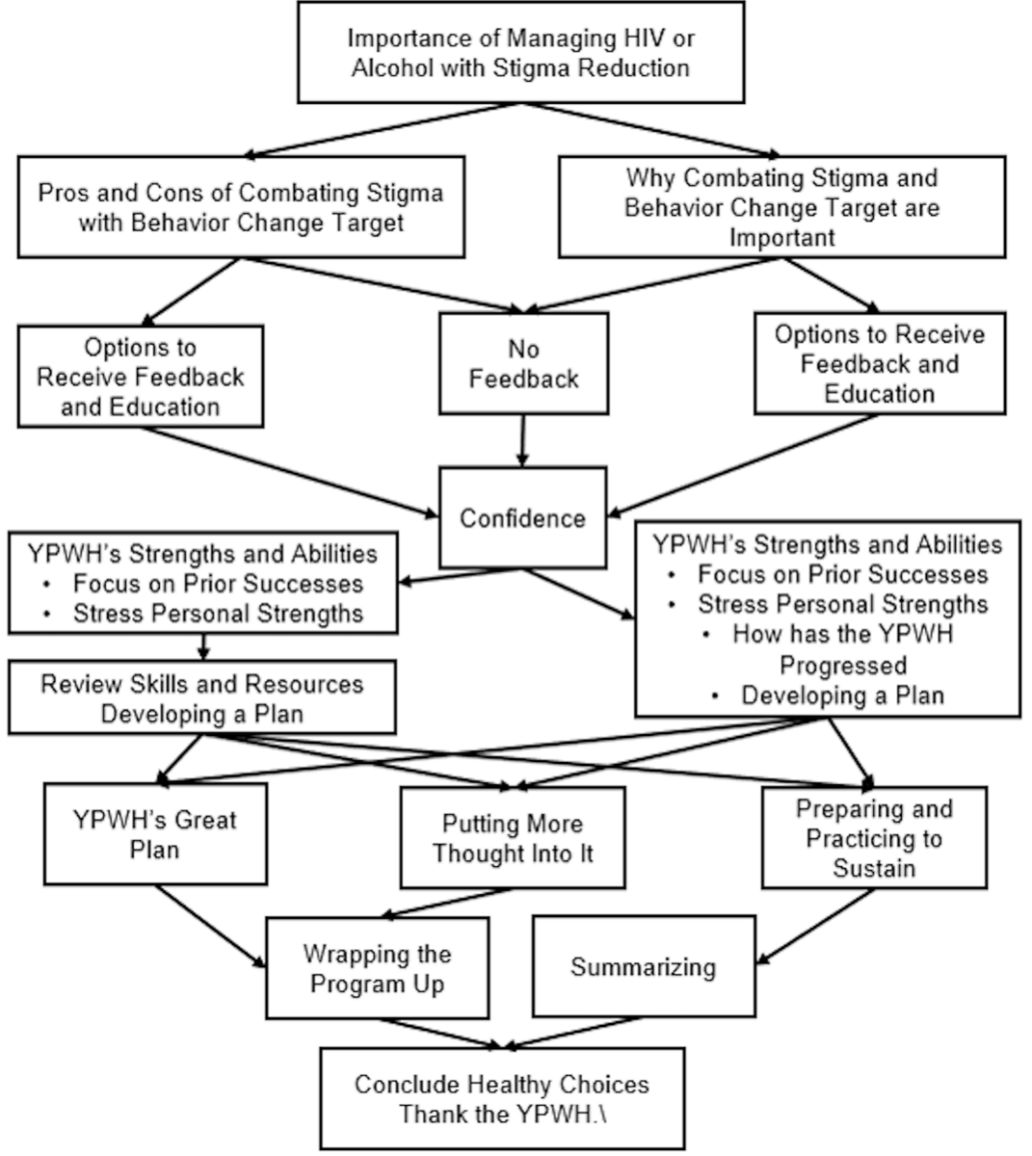
Flow of the Healthy Choices intervention across the 4 sessions. YPWH: young person with HIV.

Healthy Choices participants will receive 4 approximately 30-minute sessions over 2 months in community settings selected by participants. The intervention preserves the core Healthy Choices structure, whereby session 1 focuses on selecting a behavioral target related to HIV self-management or alcohol use and uses motivational interviewing to build change talk, confidence, and a personalized change plan. Sessions 2 to 4 reinforce change planning, monitor progress, and address barriers while explicitly addressing stigma and supporting autonomy. Refer to Figure 1 for the Healthy Choices flow and process.

Control participants will receive 4 prerecorded diet and nutrition sessions matched in frequency and duration to the intervention. The study consultant delivering the control condition will not be trained in Healthy Choices to reduce contamination. This control condition is an appropriate comparison for a peer-delivered intervention focused on stigma, alcohol, and HIV self-management, because it (1) does not confound the effects of the intervention and (2) enables disentanglement of time-attention effects from those of the Healthy Choices intervention. We selected diet and nutrition sessions, specifically because there is an ongoing need to improve the overall health status of young people with HIV, and these areas are public health priorities in the Dominican Republic. At the end of the 6-month follow-up period, control participants will be offered the intervention.

### Measures

Primary outcomes are feasibility and acceptability. Secondary and exploratory outcomes will evaluate stigma, HIV-related outcomes, alcohol and other substance use, and selected psychosocial constructs thought to influence intervention effects (Table 1) [18-26].

**Table 1:** Aim 3 outcome measures.

Constructs	Assessment time points	Measures
Acceptability	Baseline and follow-up	Acceptability of Intervention Measure; enrollment target ≥80%
Feasibility	1 month and follow-up	Feasibility of Intervention Measure; implementation feasibility; recruitment and retention; session attendance
Viral load	Baseline and 1, 3, and 6 months	Electronic health record extraction and self-report
Antiretroviral adherence	Baseline and 1, 3, and 6 months	Antiretroviral adherence questionnaire
HIV-related stigma	Baseline and 1, 3, and 6 months	HIV Stigma Scale
Other stigmas	Baseline and 1, 3, and 6 months	Substance-related stigma items
Alcohol and other substance use	Baseline and 1, 3, and 6 months	Alcohol Use Disorders Identification Test–Consumption and Alcohol, Smoking and Substance Involvement Screening Test
Demographic characteristics	Baseline	Age, nativity, education, and income
Social support and loneliness	Baseline and 1, 3, and 6 months	Social Provisions Scale; University of California, Los Angeles Loneliness Scale
Positive affect	Baseline and 1, 3, and 6 months	Positive and Negative Affect Schedule–positive affect
Coping	Baseline and 1, 3, and 6 months	Coping With Stigma Measure
Depression	Baseline and 1, 3, and 6 months	9-item Patient Health Questionnaire

### Data Collection and Data Management

Self-report data will be collected at baseline and at 1-, 3-, and 6-month follow-up assessments using Research Electronic Data Capture (REDCap; Vanderbilt University) or Qualtrics (Qualtrics Inc), accessed by a secure link and compatible with computers, tablets, and mobile devices. Viral load information will be obtained during the consent process by collecting the participant’s HIV clinic information and obtaining permission to contact the clinic for the most recent prior viral load and viral load values at 1, 3, and 6 months after completion of the intervention or control condition.

Datasets will be stored on encrypted, password-protected systems approved by the institutional review board. Spanish transcripts will be translated into English for analysis. Quantitative data will be exported to SAS (version 9.4; SAS Institute Inc) software for statistical analysis.

### Sample Size and Hypotheses

Because this is a pilot study, the trial is not powered to detect definitive intervention effects. The target sample size is 45 young people with HIV, representing approximately 15% of the estimated sample needed for a future fully powered trial. A future 1:1 randomized controlled trial was estimated to require 130 participants per arm to detect a small-to-medium effect with 80% power and α=.05; with an estimated 85% retention rate, more than 300 young people with HIV may need to be randomized. The primary hypothesis is that the adapted intervention will be feasible and acceptable for Spanish-speaking young people with HIV. Secondary hypotheses are that intervention participants will show reductions in stigma, improvements in viral burden, and reductions in alcohol use relative to the control condition. Feasibility analyses will estimate recruitment and retention overall and by condition.

### Statistical Analysis

As a pilot study, our statistical analysis will focus on descriptive results, CIs, retention, attendance, acceptability, and ascertainment of missing data. Feasibility and acceptability thresholds will be 80%, as is standard practice in pilot studies. Group differences in retention at 3 and 6 months will be examined using chi-square tests. Depending on distributional assumptions, differences in acceptability and satisfaction will be examined with *t* tests or Mann-Whitney *U* tests. Point estimates of System Usability Scale scores of at least 70 and participant acceptability of >80% will be considered minimum criteria supporting feasibility and acceptability. Immediate and follow-up changes in stigma and related outcomes will be examined using within-group pre- and posttest analyses and between-group comparisons. Exploratory analyses will evaluate potential moderation by demographic characteristics, such as age and employment, and will explore mediation pathways to identify candidate mechanisms for a future full-scale trial. Analyses will be conducted using SAS software.

### Exit Interviews

To inform future scale-up, semi structured exit interviews will be conducted with all peer navigators who deliver Healthy Choices and with a purposively selected sample of young people with HIV randomized to the intervention arm. Interviews will assess satisfaction, burden, workflow implications, perceived value, and recommendations for implementation improvement. These interviews will be audio-recorded, transcribed, translated as needed, and analyzed using rapid qualitative analysis.

### Ethical Considerations

All participants will provide informed consent before study procedures begin. Interviews and trial activities will be conducted in private spaces to protect confidentiality. Participants will receive incentives for qualitative participation, competency-related data collection, as applicable, and follow-up assessments. The study will be conducted under the auspices of the Florida State University Institutional Review Board (STUDY00006365) and the Consejo Nacional de Bioética en Salud (ADM26-065, 002-2026). This pilot randomized controlled trial was registered at ClinicalTrials.gov on June 4, 2026 (NCT07638618). The trial was registered prior to beginning any participant enrollment.

## Results

At the time of manuscript preparation (ie, during the study start-up period), partnership development, key-informant consultation, and conceptual preparation for adaptation had already occurred. The study is structured as a 36-month project with sequential qualitative exploration, intervention adaptation and translation, peer navigator training, pilot randomization, and follow-up assessments. The qualitative and adaptation phases are expected to precede pilot testing, followed by follow-up assessments at 1, 3, and 6 months for trial participants. Final results are expected after completion of follow-up assessments and data analysis.

## Discussion

### Anticipated Findings

This protocol describes a community-engaged and implementation science–guided strategy for adapting a previously effective intervention to a new linguistic and sociocultural context. The study responds to a clear gap: despite the central role of stigma, alcohol use, and poor HIV self-management in shaping outcomes among young people with HIV, few interventions address these concerns together, and even fewer still are designed for Spanish-speaking young adults in the Caribbean with direct relevance to US HIV care priorities. An innovative feature is the coupling of intervention adaptation and implementation preparation using implementation science models within the same trial to accelerate the translational pipeline. This design-for-dissemination approach is efficient and can lead to quicker adoption if successful.

### Anticipated Contributions

This protocol is expected to yield 3 main contributions. First, it will generate contextually grounded knowledge about barriers, priorities, and implementation strategies for serving young people with HIV in community settings in the Spanish-speaking Dominican Republic. Second, it will produce an adapted Spanish-language version of Healthy Choices with corresponding fidelity and training procedures. Third, it will provide pilot data on feasibility, acceptability, and early outcome signals needed to justify a future hybrid type 1 effectiveness-implementation trial [27]. Major strengths include the use of the EPIS framework to structure implementation inquiry, the use of ADAPT-ITT for systematic intervention adaptation, engagement of CBO partners and the YAB throughout the project, training and fidelity procedures for community navigators, and a randomized pilot design with both quantitative and qualitative evaluation.

### Limitations

As a pilot study, the trial is not powered to test efficacy definitively, and some outcomes, such as alcohol use, rely on self-report. Viral load ascertainment depends on clinical records rather than on direct biological specimen collection. In addition, adaptation decisions may need to reconcile countervailing stakeholder preferences. These limitations are appropriate for the aims of a pilot protocol and are intended to inform the design of a future full-scale trial.

### Conclusions

If the adapted intervention proves feasible and acceptable, this work will provide a strong foundation for a larger trial of the Spanish-language Healthy Choices intervention in the Dominican Republic, the United States, or other high-priority Spanish-speaking settings. More broadly, the study may inform how evidence-based interventions can be adapted with youth and community partners to address stigma and HIV outcomes in ways that are both developmentally responsive to emerging adulthood and informed by implementation science.

## Appendix

Multimedia Appendix 1 Peer review report from the Fogarty International Center of the National Institutes of Health under Award Number R01TW013191.

## Abbreviations

ADAPT-ITT: assessment, decision, adaptation, production, topical experts, integration, training, and testing
CBO: community-based organization
EPIS: exploration, preparation, implementation, and sustainment
REDCap: Research Electronic Data Capture
YAB: youth advisory board
