# Supplementary material for: Healthy Choices Intervention to Address Stigma, Alcohol Use, and Self-Management in Spanish-Speaking Young People With HIV: Protocol for a Pilot Randomized Controlled Implementation Study
Source: JMIR Res Protoc. 2026 Jul 29;15:e103611. doi: 10.2196/103611 (PMC13469826; doi:10.2196/103611)
Supplement: Supplementary file 1 [file resprot_v15i1e103611_app1.pdf]

SUMMARY STATEMENT

(Privileged Communication)

PROGRAM CONTACT:
GEETHA Bansal
(301) 496-1653
geetha.bansal@nih.gov

Release Date: 05/15/2025
Revised Date:

Application Number: 1R01TW013191-01

Principal Investigators (Listed Alphabetically):

BUDHWANI, HENNA (Contact)
WATERS, JOHN

Applicant Organization: FLORIDA STATE UNIVERSITY

Review Group: ZRG1 CCHI-L (56)
Center for Scientific Review Special Emphasis Panel
PAR: HIV/AIDS Behavioral Interventions and Health Outcomes
AIDS - EXP. REV.

Meeting Date: 04/23/2025
Council: MAY 2025
Requested Start: 07/01/2025
Opportunity Number: PAR-23-190
PCC: HIVSTIG
Dual IC(s): DE, DK, HD, MH

Project Title: Healthy Choices to Reduce Stigma and Improve Self-Management of Alcohol and HIV among Young Adults
SRG Action: Impact Score:29 Percentile:12 #
Next Steps: Visit https://grants.nih.gov/grants/next_steps.htm
Human Subjects: 48-At time of award, restrictions will apply
Animal Subjects: 10-No live vertebrate animals involved for competing appl.
Participant Sex: 1A-Both sexes, scientifically acceptable
Participant Race and Ethnicity: 5A-Only foreign subjects, scientifically acceptable
Participant Age: 7A-Only Adults, scientifically acceptable

Project Year	Direct Costs Requested	Estimated Total Cost
1	150,000	213,538
2	125,000	177,948
3	125,000	177,948
TOTAL	400,000	569,434

1R01TW013191-01 Budhwani, Henna

PROTECTION OF HUMAN SUBJECTS UNACCEPTABLE

ADMINISTRATIVE NOTE

RESUME AND SUMMARY OF DISCUSSION: The proposed research will assess an adaptation of Healthy Choices, a four-session behavior change communication intervention, to reduce substance use, HIV stigma, viral load, and depression in emerging adults in the Dominican Republic. The rigor of the prior research is strong, and the project's high significance lies in its ability to advance what is currently known about substance use, stigma, and viral suppression in young adults and lead to future full-scale interventions for underserved, Spanish-speaking young adults with HIV. This is a highly successful investigative team with the necessary expertise in HIV stigma, implementation science, and biostatistics to achieve the proposed aims of the study. The project was considered highly innovative with reviewers citing the inclusion of intersectional stigma and use of communication science techniques as score-driving strengths. The panel noted numerous strengths of the approach including the focus on implementation throughout the proposal, iterative development and testing, protocols for resolving differences between the participants and investigators for adaptation, use of theoretical frameworks, emphasis on community-engagement, and inclusion of capacity-building activities to facilitate future uptake. The panel also noted weaknesses identified in the approach including limited details on stigma-related eligibility criteria, justification for timing of follow-up assessments, and attention to alcohol use. There were also concerns around human subjects protections. Following discussion, the weaknesses were viewed as minor and addressable. The proposed project was seen as potentially very important with a high probability of success. Overall, this was an impressive application from an exceptional group of investigators and enthusiasm was very high. Findings are expected to have a high impact on the field of HIV stigma, substance use, and viral suppression among young adults in the Dominican Republic.

DESCRIPTION (provided by applicant): Healthy Choices is a four-session behavior change communication intervention that was developmentally tailored for emerging adults to address self-management of risk behaviors and HIV with evidence of positive effect on stigma and depression, built on Motivational Enhancement Therapy, integrating Motivational Interviewing with brief cognitive-behavioral strategies. Healthy Choices can be delivered in community settings by trained community health workers. When delivered with fidelity and in adequate dose, Healthy Choices results in reductions in substance use, HIV stigma, viral loads, and depression over follow-up compared to standard care. In the Dominican Republic, stigmas harm YPWH. The Dominican Republic is a low- to middle- income country in the Latin America and Caribbean region, is 1 of 5 countries that accounts for over 95% of Caribbean HIV infections, has an 11% rate of HIV in key populations, and a culture that perpetuates stigmatizing attitudes and behaviors toward YPWH. To our knowledge, there are no Spanish-language interventions that concurrently address mental health, viral suppression, and stigma, tailored for young adults who are in a developmental period marked by exploration, risk-taking, and a need for autonomy in health decision making. Considering the need for stigma reduction among YPWH to improve rates of viral suppression, reduce poor mental health, and encourage healthy coping by reducing substance use, we propose to adapt Healthy Choices for Spanish with local contexts plus co-create implementation strategies with community advising for future scale up. We will pilot test the adapted intervention and proposed intervention strategies, using a community-led implementation approach for feasibility, acceptability, and to assess for a signal of potential effectiveness on continuum of care outcomes including antiretroviral adherence and viral load. Considering the importance of context and the community-orientation of this study, we will apply the Exploration, Preparation, Implementation, Sustainment (EPIS) implementation science model, focusing on the Exploration and

Preparation phases. We will engage five community-based organizations (CBO) and one clinic in the Dominican Republic that work extensively with YPWH. Our international investigator team has a long history of successful collaboration with impact on policy in the Latin American and Caribbean region. We propose three aims: (1) Elucidate barriers and implementation strategies for the Healthy Choices intervention; (2) Adapt and culturally translate the intervention for local contexts, and (3) Pilot test Healthy Choices with implementation strategies for feasibility and acceptability. If successful, we will have preliminary data for a full-scale hybrid type 1 effectiveness-implementation randomized controlled trial of the Healthy Choices intervention for underserved Spanish-speaking YPWH.

PUBLIC HEALTH RELEVANCE: Considering the need for stigma reduction among young people with HIV (YPWH) to improve rates of viral suppression, reduce poor mental health, and encourage healthy coping by reducing substance use in the Dominican Republic, we propose to adapt the Healthy Choices intervention for Spanish with local contexts plus co-create implementation strategies with community advising for future scale up. We will pilot test the adapted intervention and proposed intervention strategies, using a community-led implementation approach for feasibility, acceptability, and to assess for a signal of potential effectiveness on continuum of care outcomes including antiretroviral adherence and viral load. Considering the importance of context and the community-orientation of this study, we will apply the Exploration, Preparation, Implementation, Sustainment (EPIS) implementation science model, focusing on the Exploration and Preparation phases.

CRITIQUE 1

Significance: 2
Investigator(s): 2
Innovation: 3
Approach: 3
Environment: 1

Overall Impact: This proposal aims to adapt the Healthy Choices intervention, which addresses stigma and aims to improve HIV care continuum outcomes, for young people with HIV (YPWH) in the Dominican Republic. The adaptation will focus on language translation, necessary changes to facilitate community-based delivery, and content-specific changes relevant to YPWH in the Dominican Republic. The investigator team is strong with a history of stigma reduction interventions, local connections in the Dominican Republic, and creation of the original Healthy Choices intervention. The overall study design is well-grounded in multiple frameworks, including EPIS and ADAPT-ITT. There is good community buy-in and enthusiasm for the project, with 5 CBOs indicating their support and willingness for their peer navigators to participate in adapting and delivering the intervention. Overall, there are few minor to moderate weaknesses noted. One is that the study aims to assess effects on viral load and substance use, but there are no inclusion criteria specifying that participants need have high viral load and/or high levels of substance use, which may make it difficult to see any effects on those outcomes. Another significant weakness is that there is a lack of discussion around the alcohol and/or substance use outcomes, despite a central focus of the original intervention being on reducing alcohol use. Other weaknesses noted are relatively minor. Overall, this is a well-thought-out proposal that is rigorous, engages the community, and has potential to have positive effects on care continuum outcomes among YPWH in the Dominican Republic.

1. Significance:

Strengths

- The Dominican Republic has a high burden of HIV, especially among young people
- Healthy Choices has been shown to be an effective and acceptable intervention for YPWH, so it is good science to use that framework instead of developing an entirely new intervention
- There is a need for Spanish-language versions of stigma reduction interventions, particularly in light of high levels of HIV stigma in Latin and Caribbean countries

Weaknesses

- More info is needed on current care continuum outcomes (e.g., % engaged in care, % virally suppressed) among young people with HIV in the Dominican Republic to help justify the need for the study

2. Investigator(s):

Strengths

- Dr. Budhwani is an expert in the development and delivery of HIV stigma interventions
- Dr. Waters has a longstanding collaboration with Dr. Budhwani, has experience with HIV stigma research, and serves as the local PI for the participant population
- Inclusion of Dr. Naar, the creator of the Healthy Choices intervention, adds considerable rigor and expertise to the proposed project
- All investigators have a strong track record of collaborating together

Weaknesses

- It is not explicitly stated if investigators have experience in Rapid Qualitative Analysis, which could be problematic given its frequent use in the proposed project

3. Innovation:

Strengths

- Planned inclusion of intersectional stigmas adds an innovative spin to the existing intervention
- The Dominican Republic has not often been included in NIH-funded intervention studies, despite high burden of HIV

Weaknesses

- Face-to-face stigma reduction interventions are not particularly innovative; inclusion of at least some technological aspects may have helped to increase relevance/acceptability for a young adult population

4. Approach:

Strengths

- The study is guided by clear frameworks at multiple phases of the adaptation process, including EPIS, the Health Stigma and Discrimination Framework, and ADAPT-ITT
- The proposed adaptation process is rigorous and provides ample opportunity for community partners and members of the target population to provide feedback on each iteration of the intervention
- The use of an existing intervention (Healthy Choices) that has demonstrated effectiveness in community settings adds to the rigor of the overall intervention approach

- Use of Rapid Qualitative Analysis is appropriate given the timeline of the proposed study
- 2:1 randomization is acceptable given the timeline and focus on evaluating acceptability and feasibility

Weaknesses

- Key study hypotheses are related to reductions in viral load and substance use; however, there are no inclusion criteria stipulating that participants need to have less than optimal viral load or engage in high levels of substance use. If participants are virally suppressed and not using alcohol/substances often, there may be little variation on those outcomes.
- The proposal discusses both substance use and alcohol use interchangeably, but there seems to be more of a focus on alcohol use given that in Healthy Choices the participants can choose to focus on medication adherence or alcohol use alongside stigma. It's not clear if the intervention is going to be modified to address multiple types of substances or will remain focused on alcohol use. Overall, more explication of the role of substance use in the intervention and assessment of outcomes is needed.
- There is no mention of compensation for the Youth Advisory Board, which is concerning given that they are expected to meet monthly.
- Only 3-6 intervention participants will be invited to complete exit interviews, which seems like too small of a sample to actually get useful qualitative information.
- Aim 1 requires 8-12 peer navigators to participate; it is not clear how many are employed at the participating CBOs. Information on the overall pool of peer navigators would be helpful to establish feasibility of recruitment (minor)
- "Substance abuse" is listed as a categorical outcome, but it is not clear how this will be operationalized given the use of scales like the AUDIT-C and ASSIST. A more detailed measure that captures quantity/frequency of use of alcohol and substances may be more appropriate given the focus of the intervention.

5. Environment:

Strengths

- FSU has a strong track record of supporting HIV-related research
- CVCC has necessary resources to support local implementation of the research in the Dominican Republic.

Weaknesses

- None noted by reviewer.

Study Timeline:

Strengths

- While the timeline seems tight, it is likely feasible given the investigators' existing connections, buy-in from CBOs, and prior work in this area

Weaknesses

- Recruitment rate from prior studies with YLPH would help to establish feasibility of recruiting the entire sample in 6 months

Protections for Human Subjects:

Unacceptable Risks and/or Inadequate Protections

- More detail is needed on how the peer navigators will be protected jobwise if they choose to opt-out or if they are unable to achieve fidelity in the intervention. While it is stated that they will be told that their participation will not affect their employment, more assurances/protections would be useful.

Data and Safety Monitoring Plan (Applicable for Clinical Trials Only):

Acceptable

- Plan is acceptable

Inclusion Plans:

- Sex: Distribution justified scientifically
- Race and Ethnicity: Distribution justified scientifically
- For NIH-Defined Phase III trials, Plans for valid design and analysis: Not applicable
- Inclusion/Exclusion Based on Age: Distribution justified scientifically
- Target population is well-justified based on prior research and epidemiologic trends

Vertebrate Animals:

Not Applicable (No Vertebrate Animals)

Biohazards:

Not Applicable (No Biohazards)

Resubmission:

Renewal:

Revisions:

Applications from Foreign Organizations:

Justified

- Justified based on rates of HIV in the Dominican Republic

Resource Sharing Plans:

Acceptable

- Plan is acceptable.

Budget and Period of Support:

Recommend as Requested

CRITIQUE 2

Significance: 2
Investigator(s): 1
Innovation: 3
Approach: 2
Environment: 1

Overall Impact: This is an excellent proposal that is rigorous, well conceptualized, detailed, with a strong scientific premise. This study proposes to adapt and pilot test the Healthy Choices intervention for Spanish and for young people with HIV in the Dominican Republic and identify implementation strategies for future scale up. Healthy Choices in an evidence-based intervention that has been effective at reducing viral burden, alcohol use, and stigma in young people in the US. The goal of the current study is to ultimately prepare for a full-scale hybrid type 1 effectiveness-implementation randomized controlled trial of the Healthy Choices intervention for underserved Spanish-speaking YPWH. The team is well established each with unique roles on the project, and an established history. The proposed intervention to be adapted from Healthy Choices is appropriate for this context with its community-based approach to stigma. Addressing implementation barriers from the start of the very start of the study is a strength that will likely result in a sustainable community-informed intervention and the plans to adapt, test, and address implementation barriers is rigorous. A few smaller points are raised about measurement and considerations for the role of alcohol use and related stigma. Overall, this is an outstanding proposal with the potential for high impact.

1. Significance:

Strengths

- Young people with HIV have challenges with self-management as evidenced by high rates of consumption of alcohol and other drugs and low rates of viral suppression.
- The Dominican Republic has some of the highest HIV rates in the region and a culture that stigmatizes sexual and gender minority populations with HIV.
- Few stigma interventions exist for this population and community-based approaches outside of the clinic may less stigmatizing.
- A strong case is made for why the Healthy Choices intervention is a suitable intervention to address these issues in the Dominican Republic, given its efficacy, design (community focused), and flexibility.

Weaknesses

- Stigma is described as a problem among YPWH, including in the DR, however, there are no statistics presented on the prevalence of stigma from patient and provider perspectives, nor data on the different types of stigma at play (HIV and other types of stigma). It would be informative to understand more about the extent of the problem in this setting given that the intervention hinges on that premise.

2. Investigator(s):

Strengths

- This is a highly accomplished team with complementary roles and skill sets.

- MPI Budhwani is a medical sociologist with implementation science expertise.
- MPI Waters is a scientist based in the DR and has been collaborating with the team for a decade.
- Co-I Naar and Wang also add relevant expertise to the project in terms of the Healthy Choices intervention (Naar) and biostatistics for clinical trials (Wang).

Weaknesses

- None noted by reviewer.

3. Innovation:

Strengths

- Minor innovations are noted around the use of novel communication science techniques for behavior change and use of videos for training.

Weaknesses

- Adaptation of Healthy Choices to the DR context will produce a new intervention but is not necessarily challenging the status quo in terms of innovation.

4. Approach:

Strengths

- The flow of the aims and data collected (starting with implementation at the start) ensures that implementation issues are center-stage and considered early on.
- A rigorous process is in place to resolve diverging feedback from participants on the implementation and adaptation decisions.
- Capacity-building activities will help build infrastructure to facilitate future uptake, if found feasible/acceptable in this study.

Weaknesses

- Unclear whether selected facilitators (who participated in earlier stages) will be told in advance about potential future roles, as that may impact responses provided in hopes to being viewed positively by the investigators.
- Eligibility criteria around "self-reports of stigma" is not clear in terms of how this will be measured and how decisions are made about level of stigma to qualify—presumably this isn't measured with a 'yes' or 'no'.
- Rationale for conducting three follow up assessments in a relatively short time frame (6 months) is not provided.
- Unclear whether stigma measures (in particular) have been culturally vetted for DR, adequately translated into Spanish, and are valid/reliable in this setting.
- Attention to alcohol use is highlighted in the first sentence of the proposal (significance), however, attention somewhat decreases throughout the proposal. It is not clear how important alcohol use should be in the intervention and trial design. For example, it was surprising to not see more rigorous measures used such as timeline follow-back or analysis plans to parse out the role of alcohol/drug use and explore stigma around use.

5. Environment:

Strengths

- Resources and infrastructure at FSU, UMass, and the CVC will provide ample support for this study.

Weaknesses

- None noted by reviewer.

Study Timeline:

Strengths

- The timeline is reasonable to accomplish the three study aims.

Weaknesses

- None noted by reviewer.

Protections for Human Subjects:

Acceptable Risks and/or Adequate Protections

- Protections in place for risks are appropriate.

Data and Safety Monitoring Plan (Applicable for Clinical Trials Only):

Acceptable

- A DSMB will be established for safety monitoring and plans are clear.

Inclusion Plans:

- Sex: Distribution justified scientifically
- Race and Ethnicity: Distribution justified scientifically
- For NIH-Defined Phase III trials, Plans for valid design and analysis: Not applicable
- Inclusion/Exclusion Based on Age: Distribution justified scientifically

Vertebrate Animals:

Not Applicable (No Vertebrate Animals)

Biohazards:

Not Applicable (No Biohazards)

Resubmission:

Renewal:

Revisions:

Applications from Foreign Organizations:

Justified

- Justification provided is appropriate.

Resource Sharing Plans:

Acceptable

Authentication of Key Biological and/or Chemical Resources:

Acceptable

Budget and Period of Support:

Recommend as Requested

CRITIQUE 3

Significance: 1
Investigator(s): 1
Innovation: 2
Approach: 3
Environment: 1

Overall Impact: This study team seeks to adapt an existing intervention—Healthy Choices—for young people with HIV in the Dominican Republic, a primarily Spanish-speaking low to middle income country. The team asserts that there are currently no Spanish language interventions that address mental health, viral suppression and stigma tailored for young adults. This intervention is based on Motivational Enhancement Theory and integrates motivational interviewing with brief cognitive behavioral strategies supported by the Health Stigma and Discrimination Framework. The study focuses on a significant problem—HIV care engagement and substance use among young people—in a region heavily impacted by HIV but less-often the focus of research of this type. It has a strong scientific premise and will build on an existing tested and effective intervention shown to reduce stigma and substance use and increase engagement in HIV care. The study is innovative in its application of cultural adaptation strategies in this context, its focus on implementation in the community rather than the clinic setting, its aim to speak to the unique stigma context of the DR, and its approach to coaching and providing feedback to those delivering the intervention. The study team and research environment are well-prepared to implement this study and have a history of collaboration that will support successful completion of the research. The research design is strong, relying on the EPIS implementation science framework for implementation and sustainability strategies and the ADAPT-ITT adaptation framework for intervention content changes. Enthusiasm for the study was limited by three key concerns: the choice to translate qualitative data from Spanish to English and then conducting analyses rather than conducting analyses in Spanish and then translating the results, the lack of detail regarding the quantitative measures to be employed, and glossing over the issue of the key migrant population (Haitians) who may not be fluent in Spanish and for whom Kreyol versions of materials would be beneficial.

1. Significance:

Strengths

- Young people often have issues with executive function and often take more risks than older people, leading to increased risk behavior including substance use and sexual risk behaviors. Those young people with HIV often see increased risk behaviors. This increases the chances of HIV transmission and substance use disorder. In high HIV prevalence areas (such as the Dominican Republic), this is further increased.
- A range of intersectional stigmas related to HIV, and substance use are particularly impactful for young adults, who have less developed coping strategies than their older counterparts, leading to suboptimal outcomes relative to HIV and substance use.
- This study aims to culturally adapt an existing intervention, Healthy Choices, that has been tailored for young people with HIV and has demonstrated improvements in viral load, substance use, and stigma trajectories in a full-scale multi-site RCT.
- The DR is one of five nations that are responsible for 95% of HIV infections in the Latin America and Caribbean region. It has a culture that stigmatizes people with HIV as well as sexual minorities. Such stigmas further limit work to improve health outcomes relative to HIV. These stigmas are also modified by further intersectionality: migrant status, sex work, religion, substance use, age, and socioeconomic status. In particular they limit care engagement.
- Clinic-based interventions may not reach young people as stigma reduces engagement with healthcare systems. Community-based interventions implemented alongside clinic-based interventions allow young people to choose where and how they want to engage. Healthy Choices is a four-session intervention that can be implemented in community settings.
- Key features of Healthy Choices—Motivational Enhancement Therapy and Motivational Interviewing—have been shown to reduce stigma, resulting in better engagement in care and reduced substance use.
- Building in implementation science assessment, via the EPIS model from the beginning of the RCT better situates the intervention for successful implementation and sustainability.

Weaknesses

- None noted by reviewer.

2. Investigator(s):

Strengths

- This study team has the experience and expertise required to conduct this study successfully.
- The contact PI has expertise in stigma intervention research and implementation science in global settings. Additional expertise in mental health disparities, preventative health (in particular sexual and reproductive health), HIV treatment and prevention, intersectional stigma and implementation science.
- The MPI has expertise in participatory action research and community engaged research as well as regional expertise.
- The remaining team members bring expertise in the Healthy Choices intervention (one is the creator of the intervention), motivational interviewing (particularly in the context of HIV), the EPIS adaptation framework and implementation science more broadly, RCT design and data analysis (with a focus on machine learning approaches), and adolescent risk behavior.

Weaknesses

- None noted by reviewer.

3. Innovation:

Strengths

- The study applies innovative approaches to cultural tailoring that have resulted in culturally specific motivational statements known to impact stigma, such as overt communication of acceptance and autonomy support.
- The team will implement a coaching process for intervention delivery as well as a novel automated feedback process for fidelity assessment and adjustment.
- The study team will work to address the intersectional stigmas unique to the DR, rather than focusing on only one or a few, which is unusual in HIV stigma reduction interventions delivered internationally.
- LAC is the focus of relatively little research of this type.

Weaknesses

- It is unclear what ‘innovations in community engagement’ they will employ to ensure recruitment and retention of the study sample.

4. Approach:

Strengths

- A preliminary key informant meeting has gauged interest in participating in this study and sustaining the intervention after study completion, with letters of support provided from all involved entities, including community-based organizations in the DR.
- The use of EPIS in this context is justified and appropriate, guiding the integration of evidence-based practice into real-world settings.
- The Health Stigma and Discrimination Framework, which shifts the focus away from stigmatizer/stigmatized thinking and toward the broader forces that shape and perpetuate stigma, is appropriate for this context and allows participants to recognize that they are personally empowered to confront and address stigmas in their own lives regardless of the source.
- The research team as structured is well suited to conduct this study, with full engagement from a network of local community-based organizations and a youth advisory board to oversee the study.
- Preliminary studies inform the research design demonstrating the health needs for the population of interest, evidence of the efficacy of Healthy Choices in other community-based contexts, evidence of successful implementation of stigma reduction interventions in the DR, and that MI approaches can be successfully implemented.
- The formative Aim 1 is well designed and focuses primarily on the unique features of the implementation context (the DR), in particular barriers and implementation strategies that will be successful. Focus groups and interviews will target peer navigators (who will deliver the intervention) as well as young people with HIV who will receive the intervention.
- Rapid Qualitative Analysis is appropriate for the directive goals of Aim 1.
- The use of ADAPT-ITT rather than FRAME for modifying the existing intervention is justified and well suited to the aims of the study.

- The iterative approach proposed for intervention development and testing is appropriate for this study.
- The attention control focusing on diet and nutrition is appropriate, better than standard of care in this design.
- The pilot test of the intervention is grounded in Bowen's model for pilot trials and is well-designed and of an appropriate size even though not statistically powered for a full RCT.
- The hypotheses to be tested and the analytic strategy are justified and appropriate.

Weaknesses

- Conducting some of the qualitative analyses in Spanish and then translating results in addition to translating qualitative data and then conducting analyses would strengthen the validity of the qualitative analysis portions of the study.
- While migrant status will be primarily from Haiti, there is no discussion of developing a Kreyol version of the intervention and assessment materials or addressing whether these migrants will be fluent in Spanish.
- It is unclear where baseline data collection will occur in Figure 5.
- While assessment measures or noted, they are not described. Furthermore, sources and reliability/validity of measures are not provided.

5. Environment:

Strengths

- The national and international research and implementation sites have the resources and facilities necessary for the successful implementation of the study.
- The research and implementation sites also have experience in conducting similar behavioral health type studies, particularly those that focus on community engagement.
- The primary implementation site has experience working with health (in particular HIV) among vulnerable populations
- The primary implementation site has strengths in project and program monitoring and evaluation, important for this RCT.
- The two primary institutions have engaged in previous research and mentoring relationships

Weaknesses

- None noted by reviewer.

Study Timeline:

Strengths

- The study timeline overall is adequate and appropriate for this three-year study.

Weaknesses

- It might be better to begin preliminary analyses for outcome before Q3 of Y3. More time might be necessary/helpful.

Protections for Human Subjects:

Acceptable Risks and/or Adequate Protections

- This behavioral health study poses limited risks and the protections are adequate.

Data and Safety Monitoring Plan (Applicable for Clinical Trials Only):

Acceptable

- The DSMP is acceptable for a behavioral study of this type.

Inclusion Plans:

- Sex: Distribution justified scientifically
- Race and Ethnicity: Distribution justified scientifically
- For NIH-Defined Phase III trials, Plans for valid design and analysis: Not applicable
- Inclusion/Exclusion Based on Age: Distribution justified scientifically
- The inclusion criteria are appropriate and justified for a study of this type focusing on young people with HIV in the DR.

Vertebrate Animals:

Not Applicable (No Vertebrate Animals)

Biohazards:

Not Applicable (No Biohazards)

Resubmission:

Renewal:

Revisions:

Applications from Foreign Organizations:

Justified

- Latin America and the Caribbean have the second highest regional rate of HIV, justifying a research focus in this region. Dominican Republic in particular is one of 5 countries responsible for 95% of HIV infections in the region.

Resource Sharing Plans:

Acceptable

Budget and Period of Support:

Recommend as Requested

THE FOLLOWING SECTIONS WERE PREPARED BY THE SCIENTIFIC REVIEW OFFICER TO SUMMARIZE THE OUTCOME OF DISCUSSIONS OF THE REVIEW COMMITTEE, OR REVIEWERS' WRITTEN CRITIQUES, ON THE FOLLOWING ISSUES:

PROTECTION OF HUMAN SUBJECTS: UNACCEPTABLE
The protection of human subjects from research risks is unacceptable. See Reviewer 1 critique. This needs to be addressed.

INCLUSION OF WOMEN PLAN: ACCEPTABLE

INCLUSION OF MINORITIES PLAN: ACCEPTABLE

INCLUSION ACROSS THE LIFESPAN: ACCEPTABLE

COMMITTEE BUDGET RECOMMENDATIONS: The budget was recommended as requested.

HUMAN SUBJECTS APPLICATIONS ADMINISTRATIVE NOTE: While NIH reviews and implements its research priorities under NIH's statutory and regulatory authorities, reviewers were instructed not to consider 'gender' when evaluating human subjects inclusions.

REVIEW CONSIDERATIONS ADMINISTRATIVE NOTE: While NIH reviews and implements its research priorities under NIH's statutory and regulatory authorities, certain sections of applications may not have been evaluated.

UNALLOWED HYPERTEXT ADMINISTRATIVE NOTE: During the review of this application, unallowed hypertext was noted in the application as only doi (digital object identifier) and .gov sites are allowed. Hypertext, hyperlinks, and URLs are only allowed when specifically noted in funding opportunities or application instructions. NIH is tracking this warning and has the authority to withdraw future noncompliant applications (NOT-OD-20-174).

Footnotes for 1R01TW013191-01; PI Name: Budhwani, Henna

Ad hoc or special section application percentiled against "Total CSR" base.

NIH has modified its policy regarding the receipt of resubmissions (amended applications).See Guide Notice NOT-OD-18-197 at https://grants.nih.gov/grants/guide/notice-files/NOT-OD-18-197.html. The impact/priority score is calculated after discussion of an application by averaging the overall scores (1-9) given by all voting reviewers on the committee and multiplying by 10. The criterion scores are submitted prior to the meeting by the individual reviewers assigned to an application, and are not discussed specifically at the review meeting or calculated into the overall impact score. Some applications also receive a percentile ranking. For details on the review process, see http://grants.nih.gov/grants/peer_review_process.htm#scoring.

MEETING ROSTER

Center for Scientific Review Special Emphasis Panel
CENTER FOR SCIENTIFIC REVIEW
PAR: HIV/AIDS Behavioral Interventions and Health Outcomes

ZRG1 CCHI-L (56)
04/23/2025

Notice of NIH Policy to All Applicants: Meeting rosters are provided for information purposes only. Applicant investigators and institutional officials must not communicate directly with study section members about an application before or after the review. Failure to observe this policy will create a serious breach of integrity in the peer review process, and may lead to actions outlined in NOT-OD-22-044 at https://grants.nih.gov/grants/guide/notice-files/NOT-OD-22-044.html, including removal of the application from immediate review.

CHAIRPERSON(S)

NYAMATHI, ADELINE M, PHD
DISTINGUISHED PROFESSOR AND FOUNDING DEAN
SCHOOL OF NURSING
UNIVERSITY OF CALIFORNIA, IRVINE
IRVINE, CA 92697

MEMBERS

ASSOUMOU, SABRINA A, MD
ASSISTANT PROFESSOR
DEPARTMENT OF MEDICINE
BOSTON UNIVERSITY SCHOOL OF MEDICINE
BOSTON, MA 02118

BARILE, JOHN PAUL, PHD
PROFESSOR
COLLEGE OF SOCIAL SCIENCES
UNIVERSITY OF HAWAII
HONOLULU, HI 96822

BARNIGHAUSEN, TILL, SCD, MD
PROFESSOR AND DIRECTOR
HEIDELBERG INSTITUTE OF GLOBAL HEALTH
UNIVERSITY OF HEIDELBERG
HEIDLEBURG, GERMANY 69120
GERMANY

BATEY, DAVID S, PHD
PROFESSOR
SCHOOL OF SOCIAL WORK
TULANE UNIVERSITY
NEW ORLEANS, LA 70112

BENNETT, ALEXANDER S, PHD
PRINCIPAL INVESTIGATOR
NATIONAL DEVELOPMENT AND RESEARCH INSTITUTES
NEW YORK, NY 10010

BOWMAN, NATALIE MCCARTER, MD
ASSISTANT PROFESSOR OF MEDICINE
SCHOOL OF MEDICINE
UNIVERSITY OF NORTH CAROLINA, CHAPEL HILL
CHAPEL HILL, NC 27516

CHANG, LARRY WILLIAM, MD
PROFESSOR
SCHOOL OF MEDICINE
JOHN HOPKINS BLOOMBERG SCHOOL OF PUBLIC HEALTH
BALTIMORE, MD 21287

CONROY, AMY ANNE, PHD
ASSOCIATE PROFESSOR
DEPARTMENT OF MEDICINE
SCHOOL OF MEDICINE
UNIVERSITY OF CALIFORNIA, SAN FRANCISCO
SAN FRANCISCO, CA 94158

FRIEDMANN, PETER D, MD
PROFESSOR
OFFICE OF RESEARCH
UNIVERSITY OF MASSACHUSETTS
MEDICAL SCHOOL - BAYSTATE
SPRINGFIELD, MA 01199

GREEN, HAROLD D JR, PHD
ASSOCIATE PROFESSOR
DEPARTMENT OF APPLIED HEALTH SCIENCE
SCHOOL OF PUBLIC HEALTH
INDIANA UNIVERSITY BLOOMINGTON
BLOOMINGTON, IN 47405

HEFFRON, RENEE A, PHD
PROFESSOR AND DIRECTOR
DEPARTMENTS OF MEDICINE
CENTER FOR AIDS RESEARCH
UNIVERSITY OF ALABAMA AT BIRMINGHAM
BIRMINGHAM, AL 35233

HORVAT DAVEY, CHRISTINE, PHD
ASSISTANT PROFESSOR
FRANCES PAYNE BOLTON SCHOOL OF NURSING
CASE WESTERN RESERVE UNIVERSITY
CLEVELAND, OH 44106

KUHNS, LISA MARY, PHD
RESEARCH ASSOCIATE PROFESSOR
DEPARTMENT OF PEDIATRICS
FEINBERG SCHOOL OF MEDICINE
NORTHWESTERN UNIVERSITY
CHICAGO, IL 60612

LAUCKNER, CAROLYN, PHD
ASSISTANT PROFESSOR
DEPARTMENT OF BEHAVIORAL SCIENCE
UNIVERSITY OF KENTUCKY
LEXINGTON, KY 40536

MOITRA, ETHAN, PHD
ASSOCIATE PROFESSOR
DEPARTMENT OF PSYCHIATRY AND HUMAN BEHAVIOR
ALPERT MEDICAL SCHOOL
BROWN UNIVERSITY
PROVIDENCE, RI 02906

SEROVICH, JULIANNE M, PHD
PROFESSOR AND DEAN
COLLEGE OF BEHAVIORAL AND COMMUNITY SCIENCES
UNIVERSITY OF SOUTH FLORIDA
TAMPA, FL 33620

SIGEL, KEITH MAGNUS, MD
PROFESSOR
DEPARTMENT OF MEDICINE
DIVISION OF GENERAL INTERNAL MEDICINE
MOUNT SINAI SCHOOL OF MEDICINE
NEW YORK, NY 10029

STEWART, JENELL, DO
ASSISTANT PROFESSOR
DEPARTMENT OF INTERNAL MEDICINE
HENNEPIN HEALTHCARE/HCMC
MINNEAPOLIS, MN 55415

THAMES, APRIL D, PHD
PROFESSOR
DEPARTMENT OF PSYCHIATRY
UNIVERSITY OF CALIFORNIA, LOS ANGELES
LOS ANGELES, CA 90095

WILLIAMS, MICHELLE S, PHD
ASSOCIATE PROFESSOR
DEPARTMENT OF GLOBAL AND COMMUNITY HEALTH
GEORGE MASON UNIVERSITY
FAIRFAX, VA 22030

SCIENTIFIC REVIEW OFFICER

PAGE, LINDSEY LEE
SCIENTIFIC REVIEW OFFICER
THE CENTER FOR SCIENTIFIC REVIEW
THE NATIONAL INSTITUTES OF HEALTH
BETHESDA, MD 20892

EXTRAMURAL SUPPORT ASSISTANT

MACPHERSON, SETH LOPAKI
EXTRAMURAL SUPPORT ASSISTANT
CENTER FOR SCIENTIFIC REVIEW
NATIONAL INSTITUTES OF HEALTH
BETHESDA, MD 20892

Consultants are required to absent themselves from the room during the review of any application if their presence would constitute or appear to constitute a conflict of interest.
